# Soluble CD40 ligand and prolactin levels in migraine patients during interictal period

**DOI:** 10.1007/s10194-011-0306-8

**Published:** 2011-02-18

**Authors:** Sibel Guldiken, Baburhan Guldiken, Muzaffer Demir, Levent Kabayel, Hulya Ozkan, Nilda Turgut, Remziye Hunkar, Selahattin Kat

**Affiliations:** 1 Department of Endocrinology, Trakya University Medical Faculty, Edirne, Turkey; 2 Department of Neurology, Trakya University Medical Faculty, 22100 Edirne, Turkey; 3 Department of Hematology, Trakya University Medical Faculty, Edirne, Turkey; 4 Department of Neurology, Social Security Hospital of Edirne, Edirne, Turkey

**Keywords:** Migraine, Prolactin, sCD40L, Subclinical atherosclerosis

## Abstract

The relationship of migraine with cardiovascular diseases has been clarified by many studies, and currently, migraine is suggested to be a systematic vasculopathy. Inflammation, thrombosis and impaired vascular reactivity are the underlying pathophysiological mechanisms of the vasculopathy. In the present study, we aimed to investigate the relationship between prolactin levels and subclinical atherosclerosis risk factors such as soluble CD40 ligand (sCD40L) and high-sensitivity CRP (hsCRP) in migraine patients during interictal period. Fifty female migraine patients and age-matched 25 female control cases were enrolled in the study. Migraine diagnosis was settled according to the ICHD-II diagnostic criteria. A questionnaire was completed about the existence of vascular risk factors. Serum samples were used to measure sCD40L, hsCRP and prolactin levels. No difference was found between the prolactin levels of the migraine patients and the controls. The sCD40L levels were significantly higher in migraine patients (*p* < 0.001). High-sensitivity CRP levels showed no difference between the groups. There was no correlation between prolactin, sCD40L, and hs-CRP levels in migraine patients. We consider that the migraine patients are prone to subclinical atherosclerosis, but this tendency is independent of prolactin levels.

## Introduction

Migraine is a kind of headache which is caused by the alterations in trigeminovascular system, but the pathophysiological mechanisms have not still been fully delineated. Today, it is known that the vascular alterations are not limited to cranial vessels, and migraine is suggested to be a systemic vasculopathy [1]. The vasculopathy of migraine is thought to reflect the endothelial dysfunction and impaired vascular reactivity. The activation of the platelets and the coagulation factors [2], the increased secretion of von Willebrand factor and tissue plasminogen activator from endothelium [3], the decrease in the circulating endothelial progenitor cells [4], which are all seen in migraine, support this theory. The demonstration of similar C-reactive protein (CRP) levels in migraineurs and healthy control subjects in recent studies [5, 6], although it had been found high in previous studies [7–9], necessitates new investigations and data about this issue.

The increasing number of studies in the past decade showed that the risk of cardiovascular disease is increased in migraine [10–13]. Cytokines and cytokine-inducible inflammatory molecules are predictors of cardiovascular events. Elevated high-sensitivity CRP (hsCRP), interleukin 6 and soluble CD40 ligand (sCD40L) levels are associated with the increased risk of cardiovascular disease [14, 15]. Soluble CD40L is a ligand of glycoprotein IIb-IIIa receptor and is involved in thrombus stabilization and platelet activation. It is a reliable predictor of cardiovascular disease [16].

Prolactin, secreted from anterior pituitary gland and meanwhile also synthesized by various immune system cells, has been reported to cause a tendency to the cardiovascular diseases. On the other hand, hyperprolactinemia induces migraine attacks [17] and also plays a role in other primary headache syndromes [18]. It is known that dopaminergic dysfunction is responsible from autonomic symptoms such as nausea and yawning [19].

In this study, we aimed to investigate the sCD40L, prolactin and hsCRP levels in migraine patients during interictal period and their relationship with migraine subtypes and attack frequency.

## Materials and methods

Fifty female migraine patients and age-matched 25 female control subjects were enrolled in the study. Migraine diagnosis was settled according to the International Classification of Headache Disorders-II diagnostic criteria [20]. Twenty-three patients had migraine with aura, while the rest had migraine without aura. The control subjects were enrolled as healthy individuals who had no headache of any kind. The study was approved by the local Ethics Committee, and all patients gave their informed consent to participate in the study. Exclusion criteria included pregnancy, disorders and drugs which affect the prolactin levels such as butyrophenones, phenothyozines, reserpine or methyldopa, history of prolactinoma, hypothyroidism, chronic renal failure and cirrhosis. A questionnaire was completed about the existence of vascular risk factors such as hypertension, diabetes, smoking and family history of cardiovascular diseases. Migraine headache attack frequency was noted as the number of attacks per month. Body mass index (BMI) was calculated on the basis of World Health Organization (WHO) recommendations.

Serum samples were collected from an antecubital vein without using a tourniquet, between 08:30 and 09:00 a.m. after overnight fasting. For hsCRP, sCD40L and prolactin, venous blood samples were centrifuged within 15 min at 3,000 rpm for 10 min, and the supernatant serum samples were transferred into polypropylene tubes at −80°C until the assays were performed. Soluble CD40L and hsCRP levels were measured by ELISA (Biosource, Immunoassay-kit, USA; DRG Inc., USA, respectively) and prolactin levels by chemiluminesense immunoassay (DPC, Immulite 2000, USA). The participants’ serum fasting glucose levels were measured using the glucose oxidase technique (Konelab 60, Finland). Cholesterol esterase enzymatic assays (Konelab 60, Finland) were used to measure total cholesterol and high-density lipoprotein-cholesterol (HDL-C). Triglyceride levels were measured by the lipase technique (Konelab 60, Finland). Low-density lipoprotein-cholesterol (LDL-C) levels were calculated according to Friedewald’s formula. Values of the platelet count and mean platelet volume were obtained from whole blood count analysis.

## Statistical analysis

All statistical analyses were performed by using SPSS 11.5 software for Windows. Data were expressed as mean ± SD. The normality of the distribution of all variables was assessed by the Kolmogorov–Smirnov test. Student’s *t* test and Pearson correlation analyses were used for normally distributed variables. The Mann–Whitney *U* test and Spearman rank correlation test were used for non-parametric variables. Logistic regression model was performed for adjusting for BMI. *P* values <0.05 were considered statistically significant.

## Results

The characteristics of the migraine patients and the control subjects are shown in Table 1. Age, anthropometric values, blood pressure, serum fasting glucose and lipid levels did not differ between the two groups. BMI was higher in the migraine group (*p* < 0.05). No difference was found between the prolactin levels of the migraine patients and the controls. The sCD40L levels were significantly higher in migraine patients (*p* < 0.001). High-sensitivity CRP levels showed no difference between the groups. There was no correlation between the prolactin, sCD40L and hs-CRP levels in migraine patients (*p* > 0.05, for all). Soluble CD40L remained significantly high in the migraine group after adjusting for BMI of both the groups (*p* = 0.002). Mean platelet volume of migraine patients was significantly higher than that of the control group (*p* < 0.05) and was correlated with sCD40L levels (*r* = 0.362, *p* < 0.05).

**Table 1:** The clinical and laboratory characteristics of all participants

	Migraine subjects (*n* = 50)	Control subjects (*n* = 25)	*p*
Age (years)	38.0 ± 8.6	34.4 ± 7.8	Ns
Hypertension (%)	9.3	9.0	Ns
Diabetes mellitus (%)	4.4	0	Ns
Smoking (%)	42.4	50	Ns
BMI (kg/m^2^)	26.54 ± 5.52	23.98 ± 3.18	<0.05
Systolic BP (mmHg)	118.09 ± 17.4	123.63 ± 23.77	Ns
Diastolic BP (mmHg)	77.44 ± 16.34	77.27 ± 11.03	Ns
Glucose (mg/dl)	91.76 ± 10.76	93.85 ± 22.8	Ns
Cholesterol (mg/dl)	189.88 ± 38.04	183.42 ± 40.28	Ns
Triglyceride (mg/dl)	106.21 ± 44.54	102.85 ± 53.97	Ns
LDL (mg/dl)	119.92 ± 32.38	111.68 ± 32.90	Ns
HDL (mg/dl)	50.03 ± 12.52	49.29 ± 15.88	Ns
Platelet (/mm^3^)	274361 ± 59207	263062 ± 60698	Ns
MPV (fL)	9.53 ± 1.4	8.7 ± 0.92	<0.05
Prolactin (ng/mL)	11.34 ± 8.63	10.89 ± 5.0	Ns
hs-CRP (mg/L)	4.28 ± 4.01	3.91 ± 3.89	Ns
sCD40L (ng/mL)	11.92 ± 5.26	7.72 ± 3.6	<0.001

*Ns* non-significant, *BP* blood pressure, *MPV* mean platelet volume, *hsCRP* high-sensitivity C-reactive protein, *sCD40L* soluble CD40 ligand

No difference was found between migraine with aura and migraine without aura groups regarding the sCD40L, hsCRP and prolactin levels (Table 2). When the migraine patients were subdivided according the attack frequency, sCD40L, hsCRP and prolactin levels were indifferent among the frequent (4 or more attacks/month) and seldom (less than 4 attacks/month) attack groups (Table 3). Family history of migraine and cardiovascular disease were not associated with these parameters (*p* > 0.05, for all).

**Table 2:** Soluble CD40L, hsCRP and prolactin levels in migraineurs with aura and without aura subgroups

	Migraineurs with aura (*n* = 23)	Migraineurs without aura (*n* = 27)	*p*
sCD40L (ng/mL)	11.73 ± 4.52	12.08 ± 5.9	Ns
hsCRP (ng/L)	4.06 ± 3.98	4.46 ± 4.1	Ns
Prolactin (ng/mL)	9.45 ± 4.77	12.95 ± 10.74	Ns

*Ns* non-significant, *hsCRP* high-sensitivity C-reactive protein, *sCD40L* soluble CD40 ligand

**Table 3:** Soluble CD40L, hsCRP and prolactin levels in migraineurs with frequent (4 or more attacks/month) and seldom (less than 4 attacks/month) headache attacks

	Frequent migraine attacks (*n* = 33)	Seldom migraine attacks (*n* = 17)	*p*
sCD40L (ng/mL)	11.87 ± 4.55	14.09 ± 6.8	Ns
hsCRP (ng/L)	4.40 ± 3.72	4.76 ± 5.04	Ns
Prolactin (ng/mL)	9.70 ± 5.75	10.60 ± 3.74	Ns

*Ns* non-significant, *hsCRP* high-sensitivity C-reactive protein, *sCD40L* soluble CD40 ligand

## Discussion

The main finding in our study was the elevated sCD40L levels in migraine patients. We consider that this is a new data related to the association of migraine and vascular diseases. There are several studies reporting the association of sCD40L with cardiovascular diseases. Soluble CD40L was suggested to be a predictor for myocardial infarction and stroke [21–23]. In the studies, where the acute coronary patients were enrolled, sCD40L was found as a marker of inflammatory thrombotic activity and found related with further increased cardiovascular events [21, 22]. Recently, CD40/CD40L pathway activation and a subsequent proinflamatory milieu were reported in diseases such as obesity [24, 25], diabetes mellitus [26] and hypertension [27]. Whether migraine patients constitute a low- or high-risk group for cardiovascular disease is obscure, but high-sCD40L levels in migraine patients in our study support the presence of a vascular damage in migraine.

CD40L belongs to the tumor necrosis family and is a transmembrane protein expressed by heamatopoetic cell types such as T lymphocytes, monocytes and platelets as well as by nonheamatopoetic cells like endothelial and smooth muscle cells. Soluble form is particularly produced by platelet activation [28], is associated with plaque instability and is a predictor of the plaque complications [29, 30]. Moreover, CD40L is able to promote overexpression of tissue factor, a glycoprotein that has a crucial role in the activation of coagulation cascade [31]. In order to clarify if the increase of sCD40L in migraine patients is concordant with platelet activation, we compared between the mean platelet volume of two groups. It is a reliable marker of platelet activation such as platelet aggregation, secretion of thromboxane A2, platelet factor 4 and thromboglobulin [32, 33]. We found that the mean platelet volume of migraine patients was significantly higher than that in the controls, and it was correlated with CD40L levels in migraine. This impresses that the high sCD40L levels in our study were mainly dependent on platelet activation.

Soluble CD40L additionally has inflammatory property including expression of adhesive molecule, chemokines and metalloproteinases [29], which is different from the inflammatory pathway of CRP [21, 27]. Matrixmetalloproteinase 9, whose levels are found high during migraine attacks, degrades laminin, collagen type IV, a critical component of brain blood levels [34]. Soluble CD40L also induces the secretion of other proinflammatory cytokines such as interleukin 1 (IL-1), IL-6, IL-8, IL-10 and tumor necrosis factor (TNF) from monocytes [35], dentritic cells [36], fibroblasts [37] and epithelial cells [38]. TNF alpha, IL-6, IL1 beta and IL10 were found to be increased during migraine attacks [39]. In the present study, since we did not measure the levels of these proinflammatory cytokines, it is not possible to conclude any association of the proinflammatory property of sCD40L with the inflammation in migraine.

Another finding of our study is that the hsCRP levels of the migraineurs and the control cases were not significantly different. There are several studies reporting modest hsCRP increase in migraine patients, but this elevation was only noted in the migraine without aura group which was actually not associated with cardiovascular diseases [7–9]. A recent study carried on large number of case groups demonstrated no difference in hsCRP levels in migraine patients and non-migraineurs [5]. The elevation of hsCRP levels is a marker of inflammatory process, especially in the cardiovascular diseases, infection and malignacies. However, as known, low CRP levels can also be seen in diseases with marked tissue inflammation such as systemic lupus erythematosus [40]. Normal hsCRP value in migraine should not mean that there is no inflammation or no risk of cardiovascular disease in migraine. Other pathways, such as the inflammatory CD40 property, may be involved in the inflammatory process of migraine.

In our study, we found similar prolactin levels between the migraine and control groups during interictal period. Our results are concordant with two other studies reporting normal prolactin levels in migraine [41, 42]. It was hypothesized that the increased dopaminergic activity is responsible from prodromal, clinical and postdromal symptoms [19, 43] and, therefore, a decrease in prolactin levels should be expected during migraine attacks. Conveniently, Masoud and Fakharian [44] found a significant decrease in prolactin levels during the headache attack in migraineurs. Another study reported high prolactin levels during migraine attack, but the study was carried on subjects who had microprolactinomas [17]. Since we measured the prolactin levels only during the interictal period, we could not compare our results with these two studies.

Although prolactin has been reported to create a tendency to the cardiovascular diseases [45–48], our study demonstrated no correlation of prolactin with sCD40L and hs-CRP. It is known that increased prolactin levels in cases with prolactinoma cause obesity, insulin resistance, an increase in homocysteine levels and low-grade inflammation and subsequently a tendency to cardiovascular diseases [46, 47]. High prolactin levels stimulating sympathetic tonus cause preeclempsia in pregnancy [49]. Recently, a relationship was found between prolactin levels, blood pressure and arterial stiffness, and prolactin was accused of accelerated atherosclerosis in women with early menopause [45]. The demonstration of the intense prolactin receptor expression on advanced human coronary atherosclerotic plaques supports the role of prolactin on the development of atherosclerosis [48].

The present study demonstrated that the sCD40L levels are elevated in female migraine patients during interictal period. The small number of the groups and the omission of other hormones, such as estrogens and progesterone, which influence the biologic action of prolactin were the limitations of the study. We consider that our findings should be supported by investigations during the attack period and also in male migraineurs. Further prospective studies are needed to clarify the mechanisms underlying the association between migraine and cardiovascular diseases.
